# Supplementary material for: Hypoxemia, hypoglycemia and IMCI danger signs in pediatric outpatients in Malawi
Source: PLOS Glob Public Health. 2022 Apr 26;2(4):e0000284. doi: 10.1371/journal.pgph.0000284 (PMC10021275; doi:10.1371/journal.pgph.0000284)
Supplement: S1 Table — SpO2 results excluded from manuscript included. * Respiratory rate was assessed in 399 children. ** 3 caregiver refused SpO2-meassurment. *** No result due to refusal by caregiver (3), lack of test strips (22) and child too agitated (1). (DOCX) [file pgph.0000284.s001.docx]

S1 Table

**Supporting Table 1. IMCI general danger signs (unconscious, sleepy/lethargic, convulsing, vomits everything, unable to eat/drink) depending on clinical characteristics. SpO_2_ results excluded from manuscript included.**

|  | **IMCI general danger sign present**  N=848  n (%) | **IMCI general danger sign not present**  N=2,095  n (%) | **Total**  N=2,943  n (%) |
| --- | --- | --- | --- |
| **Respiratory signs** | | | |
| Tachypnea for age* | 38 (4.5) | 63 (3.0) | 101 (3.4) |
| RR not done/error | 718 (84.7) | 1,826 (87.2) | 2,544 (86.4) |
| Chest indrawing | 22 (2.6) | 13 (0.6) | 35 (1.2) |
| **Oxygen saturation (%)** | | | |
| Normal spO2 (94-100) | 742 (87.5) | 1,781 (85.0) | 2,523 (85.7) |
| Moderate hypoxemia (90-93) | 57 (6.7) | 126 (6.0) | 183 (6.2) |
| Hypoxemia (<90) | 21 (2.5) | 71 (3.4) | 92 (3.1) |
| Unstable to obtain a stable SpO2 wave | 28 (3.3) | 114 (5.4) | 142 (4.8) |
| No result of spO2** | 0 (0.0) | 3 (0.1) | 3 (0.1) |
| **Blood glucose concentration (mmol/l)** | | | |
| Hypoglycemia (<2.5) | 4 (0.5) | 0 (0.0) | 4 (0.1) |
| Moderate hypoglycemia (2.5-3.9) | 104 (12.3) | 197 (9.4) | 301 (10.2) |
| Normal b-glucose (4-11) | 733 (86.4) | 1,868 (89.2) | 2,601 (88.4) |
| Hyperglycemia (>11) | 5 (0.6) | 6 (0.3) | 11 (0.4) |
| No result*** | 2 (0.2) | 24 (1.2) | 26 (0.9) |
| **Nutritional status** | | | |
| Well nourished | 443 (52.2) | 1,142 (54.5) | 1,585 (53.9) |
| Moderately malnourished | 77 (9.1) | 113 (5.4) | 190 (6.5) |
| Severely malnourished | 50 (5.9) | 61 (2.9) | 111 (3.8) |
| Not known/assessed for malnutrition | 278 (32.8) | 779 (37.2) | 1,057 (35.9) |

* Respiratory rate was assessed in 399 children.

** 3 caregiver refused spO2-meassurment

*** No result due to refusal by caregiver (3), lack of test strips (22) and child too agitated (1)
