# Supplementary figures and images for: Hypoxemia, hypoglycemia and IMCI danger signs in pediatric outpatients in Malawi
Source: PLOS Glob Public Health. 2022 Apr 26;2(4):e0000284. doi: 10.1371/journal.pgph.0000284 (PMC10021275; doi:10.1371/journal.pgph.0000284)

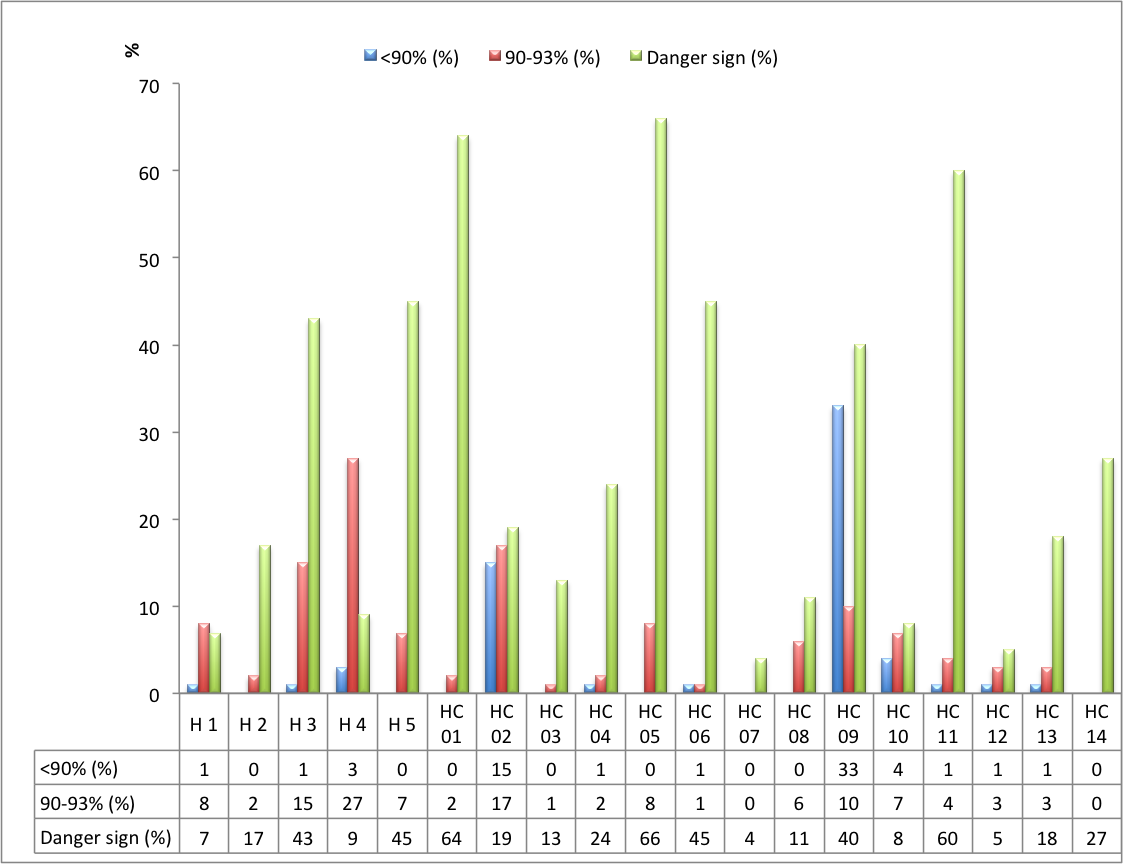

Supplement: S1 Fig — *The results of SpO2 from HC 2 and HC 9 were excluded from the analysis. (TIF) [file pgph.0000284.s002.tif]
