# Supplementary material for: Hypoxemia, hypoglycemia and IMCI danger signs in pediatric outpatients in Malawi
Source: PLOS Glob Public Health. 2022 Apr 26;2(4):e0000284. doi: 10.1371/journal.pgph.0000284 (PMC10021275; doi:10.1371/journal.pgph.0000284)
Supplement: Attachment — Submitted filename: Response to Reviewers.docx [file pgph.0000284.s003.docx]

Dear Editors

Thank you for considering our manuscript “Hypoxemia, hypoglycaemia and IMCI danger signs in pediatric outpatients in Malawi” with reference numer PGPH-D-21-00696 for publication in PLOS Global Public Health. We have revised the manuscript according to the comments from the reviewers and editorial committee. Please see our responses to the issues raised listed in bullets below.

Best wishes,

André Thunberg, MD

**Editorial comments:**

1. Please provide additional details regarding participant consent. In the ethics statement in the Methods and online submission information, please describe how verbal consent was documented and witnessed, and why written consent was not obtained.

- Additional information around consenting procedures have been added on lines 288-294.

2. In the online submission form, you indicated that "Unidentified data used in this study is available upon request from the main authors"

All PLOS journals now require all data underlying the findings described in their manuscript to be freely available to other researchers, either 1. In a public repository, 2. Within the manuscript itself, or 3. Uploaded as supplementary information.
This policy applies to all data except where public deposition would breach compliance with the protocol approved by your research ethics board. If your data cannot be made publicly available for ethical or legal reasons (e.g., public availability would compromise patient privacy), please explain your reasons by return email and your exemption request will be escalated to the editor for approval. Your exemption request will be handled independently and will not hold up the peer review process, but will need to be resolved should your manuscript be accepted for publication. One of the Editorial team will then be in touch if there are any issues.

- Data has been uploaded to the public repository Dataverse and can be accessed here: [https://doi.org/10.7910/DVN/0YWX8J](https://doi.org/10.7910/DVN/0YWX8J)

3. In your financial disclosure statement, state the initials, alongside each funding source, of each author to receive each grant.

- This information has been added.

**Reviewer #1 comments:**

*Abstract*

- Line 26: include the word “of” after “prevalence”
  - The word ”of” has been included according to your suggestion.
- Line 32: maybe you can call them research assistants instead of data collectors
  - Thank you for your suggestion; ”data collectors” has been changed to ”research assistants” throughout the manuscript.

- Line 33 and 34: include in brackets the manufacturers of the respective equipment that was used ie Lifebox LB-01 pulse oximeter and AccuCheck Aviva glucometers
  - Due to the word limits in the abstract we have chosen not to include the manufacturers in the abstract text. Please note that information about the manufacturers has been added to the methods section.
- Line 35 and 36: the author mentions severe and moderate hypoxemia and hypoglycaemia and then gives respective ranges. It is best to dissociate the moderate from severe hypoxemia or hypoglycaemia to make more clarity in the sentence
  - Please see changed text lines 35-36 for increased clarity.
- Conclusions: I am not sure whether the analysis is robust enough to draw these conclusions.
  - We feel our conclusion builds on the results as mainly mentioning the low prevlaence of severe hypoxemia and hypoglycaemia along with the issue to detect hypoxemia with current danger signs.
- The authors say that they run a chi2 test… but the outcome(s) of the study are not clear.
  - Please see table 4 and the results section discussing the differences between age groups and distribution of danger signs between those with and without hypoxemia/hypoglycaemia.

*Introduction*

- Line 60 – 62: It would be better to link the success of the IMCI program to most recent targets like the SDGs instead of the long gone MDGs
  - As IMCI has not yet been well evaluated under the SDGs we think it is relevant to mention the impact IMCI programmes had on the outcomes of the MDGs. While IMCI will need to develop to further increase its impact we believe our study findings can inform future guidelines.
- The objective of the study is not quite clear.
  - Please note that the objective of the study is stated at line 96-99;

”…we aimed to assess the prevalence of severe and moderate hypoxemia and hypoglycemia in children presenting to outpatient clinics in Mchinji district, Malawi, and to explore the overlap between different levels of hypoxemia and hypoglycemia and IMCI danger signs.”

- It would have been great to classify the IMCI in terms of the illnesses
  - While we agree that this could have been interesting, we felt the classification done by the facility health workers were not always reliable or in accordance with IMCI algorithms. For that reason, we decided to stick to a pragmatic approach by only reporting on danger signs in relation to the clinical signs and symptoms of the patient.
- Materials and methods: it would be great to describe the Emergency paediatric treatment and referral in Malawi in frontline healthcare settings – EREMISS study)study in which the current research study is nested. If the paper for this study is published then give reference to the paper. When was the EREMISS study conducted? Was the EREMISS study conducted concurrently with the study under review? What kind of participants were recruited? What was the eligibility criteria? How long was the parent study?
  - Additional information about the EREMISS study has been added on line 144-160
- Study setting: It is important to describe the different types of study sites involved in the study.. do the patients pay for the services provided? What is the structure of the healthcare setting in Malawi? How do the different study sites fit in with in the health system structure of Malawi? What kinds of patients seek health care at the different study sites in general? Do these patients pay for the services offered? What is the difference between “health centre” and “hospital”? this distinction can be described earlier on in the text so that the reader can understand the results table 1 much better.
  - Information has been added on lines 167-170.
- Include a separate section on sample size estimation in which the authors can state that all children seen in a certain time period were included.
  - As the study was nested within a bigger cohort study, we did not conduct an a priori sample size calculation for this secondary analysis. Rather, we planned for a convenience sample approach to include all sick children one day per facility over one year. However, the sample we attained is large enough to estimate the proportions of hypolycaemic and hypoxaemic children with a 1% accuracy.
- What was the rationale for only including only participants that had an acute illness? Why were those classified as having moderate, severe illness not recruited yet they could be having characteristics that may be of interest to science and policy
  - Only children coming for routine visits such as immunizations or growth controls were excluded, as stated at the end of the section ”Participant recruitment”. Consequently, children with different degrees of illness severity were included.
- Participant recruitment: line 111 – you can refer to the data collectors as research assistants.
  - ”data collectors” has been changed to ”research assistants” throughout the manuscript.
- Line 116 – 118: It is not possible that it is the children that were approached for recruitment. Actually the parents or care givers of these children are the ones who should have been approached to obtain consent for the recruitment of their children. What do the ethical guidelines say in Malawi about the assenting of children? Some of these children should have been able to assent. These procedures should be discussed in detail.
  - That is correct and now changed into ”Guardians of all children..” Regarding assent, we considered the legal age for consent as >=16 and this was above our target sample age – however, we would not insist on conducting assessments in cases where children were uncooperative (and we had options in the data collection forms for this, i.e. ‘999’ for assessments that could not be done). We have provided additional information around the consenting procedures on line 288-294.
- Data collection procedures: line 124: please include the manufacturer of the Lifebox LB-01 pulse oximeter and the machine for blood glucose, the AccuCheck Aviva point of care device.
  - The manufacturers have been added.
- How was observer bias handled while the measurements of variables was being done? This can be clarified in data collection procedures.
  - The data captured was retrieved from the patient’s health passport and the research assistans transferred the information directly to ODK. For blood glucose, the risk of bias is deemed limited. For pulse oximetry, research assistants were trained to wait for a stable curve and also had to document whether they achieved a stable curve prior to recording the saturation value – they were visited at least monthly by supervisors who checked for their performance in assessments. We therefore did not consider observer bias on the behalf of research assistants as a major concern.
- What was the qualification of the “data collectors” (research assistants)? Were they trained in handling children? Were they nurses? Midwives? Or doctors? Were they trained in research? Were the research assistants employees of the different health facilities at which the study was conducted?
  - Please see added text on lines 243-245. Research assistants were employed by the study and had no former contact/employment at the facilities where they collected data.
- Was consent sought for the collection of blood from the child (it is a separate consent process from the participation in the study)
  - Please see added info line 202-203 and 288-294.
- For those children that were hypoxemic and those that were hypoglycemic, what further steps did the study team under take to ensure that these participants received the medical care that they required? This should be highlighted for purposes of equipoise
  - The facility health workers were alerted in case of severe hypoglycemia or hypoxemia to manage the child according to standard procedures.This process was presented to the District Health Management Committee before the study started, and given the study was observational the issue of equipoise was not a concern. Please see this information on line 211-212
- Line 138: where was the data being uploaded? Was it on a server?
  - Data was uploaded to an ODK database. This has now been clarified in the manuscript.
- It would be very informative to include a section entitled variables under which the authors describe all the key / important variables that they have measured in the study ie outcome variables and exposures.
  - Please see clarification of included variables on lines 221-223.
- Line 169 – 170: the IMCI guidelines recommend the evaluation of a number of danger signs ie bacterial infection, dehydration, jaundice, diarrhoea, low blood sugar, difficulty in breathing etc. It is important for the authors to specify exactly which danger signs they included in their study and how this differed from the exposures that they objectively measured.
  - We have used the “general danger signs” as listed in the 2014 IMCI Chart booklet which only includes inability to drink/breastfeed, vomiting everything, history of convulsions/current convulsions and lethargy/unconsciousness, rather than all the clinical signs that are assessed. This is mentioned on line 215-216.
- Ethical statement: line 179 – 183: this study involved paediatric participants. The authors must show an effort to convince the readership that the participation of these participants was not coerced.
  - Additional information on consenting procedures have been added on line 288-294.
- If the data collectors were employees of the different study sites and at the same time research assistants, how did this double role play prevent coercion of participation? How was confidentiality of the information collected from the participants observed? Was the information collected by the ODK uploaded to a server? If so, how was this uploaded data handled? Was assenting for children aged 10 years or more done? What if this child declined assenting? How did the authors handle care givers or parents of children / infants that were minors (less than 18 years)? In this section the authors can also endeavour to make mention of what was done for oh how the study handled participants that needed medical attention. In the results the authors say that some parents / care givers declined participation. How were these participants handled?
  - Please note that the data collectors/rsearch assistans were not the employees of the facilities, and in order to promote a collaborative working environment for the study, we held joint meetings with the District Health Management Team, and facility managers before the study began to discuss and agree the protocol. The uploaded data was all anonymized, we did not collect information on any names or contact information. We did not have an age-threshold for caregivers providing consent for their children, given their legal status as the child’s guardian. Regarding assents, we we considered the legal age for consent as >=16 and this was above our target sample age and this was approved by the College of Medicine Research Ethical Committee. Please see added information regarding consenting procedures on line 288-294.

*Results*

- Results: was data about the father of the child collected? It would be informative to include this in the analysis as well.
  - Unfortunately, no information about the father was collected.
- What reference was used to classify the disease diagnosis? Was it the ICD? The authors can mention this.
  - The diagnosis was assigned by the facility health worker who routinely assessed the child, using their standard criteria (i.e. they should be following IMCI assessment and classification). ICD was not used and we did not verify the accuracy of the diagnoses made, as we were specifically interested in routine care.
- The authors should calculate or estimate the 95%CI for the major proportions / prevalence. This can be done in stata using the exact method.
  - We have added the confidence interval for some of the major proportions.
- The results can be presented in a more coherent manner. This section can be improved after the section of the variables is constructed.
  - We feel this comment is difficult to respond to as we are uncertain on what the reviewer wants us to change. We have chosen to present the results by main objectives, i.e. prevalence of hypoglycaemia, prevalence of hypoxemia and then prevalence of IMCI danger signs and its relation to hypoglycaemia and hypoxemia.

*Discussion*

- Discussion: this is too long and can be shortened.
  - Please see removed sections from third and fifth paragraph of discussion. We have made changes according to the overall comments from reviewers but feel uncertain in what specific way the dicussion should be made shorter – we are happy to reduce the section with specific suggestions.
- The study must be having some limitations. The authors can look into including this section in the manuscript. For example this is a purely descriptive study and it’s very difficult to make inferences to the general population.
  - Please see the key limitations, including added text, in the last paragraph of the discussion section, lines 581-592.

- THE AUTHORS SHOULD CONSIDER TO USE THE STROBE GUIDELINES WHILE REVISING THIS MANUSCRIPT
  - We have reviewed the STROBE guidelines to make sure the report adheres to the recommendations.

**Reviewer #2**

*General comments*

- This is an interesting and valuable topic.
  - Thank you.
- The data is original but there are numerous errors in the tables and the analysis is incomplete.
  - Thank you for highlighting this. We have clarified the information provided in tables.
- IMCI was developed specifically for the assessment of children below five years of age but in this study the danger signs have been applied to children up to 12 years of age without any justification or comment on the appropriateness or applicability of the danger signs to the older child. The analysis of danger signs and the relationship of these to both hypoxemia and hypoglycaemia needs to be disaggregated by age to differentiate between those under and over five years of age.
  - This is indeed a relevant comment. Please see added text in methods lines 217-220 . We have also added information about the distribution of danger signs over age groups in Table 4. We have also added information on lines 374-377 and 406-411 in results and lines 549-552 and 585-586 in discussion.
- The English is fair although there is the occasional typographical error.
  - The manuscript has been gone through again and typographical errors are corrected.

*Title*

- The title is an appropriate reflection of the content of the paper.
  - Thank you.

*Abstract*

- The abstract is a fair summary of the full content of the article.
  However it may need to be revised if further analysis reveals any differences between younger and older children.
  - As there was no difference in prevalence of danger signs between age groups we have chosen not to change the abstract info.
- Line 32 - needs to be corrected to “one day per month in each facility”
  - The sentence has been changed according to your suggestion.
- Line 34 – WHO needs to be presented in full before the abbreviation can be used
  - Considering the word limit, we chose to use the established abbreviation for WHO – but are very happy for it to be expanded if the editor allows the additional words!
- Line 42 – need to add severely ie “23.5% of the severely hypoxemic and..”
  - Severely has been added to clearify, as suggested.

*Introduction*

- The introduction is clear and succinct. It sets the scene, highlights common challenges in identifying and referring critically ill children from the primary care level and suggests a possible response to identifying these children.
  This study was undertaken to assess the validity of this suggestion.
  - Thank you!

*Method*

- Although the description of the methodology is clear it requires some expansion or additions: Justification is required for the use of IMCI danger signs out of their intended purpose ie in the assessment of children over 5 years of age. They need to provide some evidence to show that this is an appropriate tool to use in older children.
  - Please see added text on line 217-221.
- Some explanation is required as to why the authors chose to supplement the 5 IMCI danger signs with temperature, respiratory rate and chest indrawing and what evidence supports the choice of these specific signs as “danger” signs
  - Please note that we adhere to the IMCI definition of danger signs. We included chest indrawing, as this was a clinical sign which triggered referral in the 2005 IMCI guidelines, and was removed in the 2014 guidelines (for home treatment), but this decision has been questioned. It is therefore a sign to assess. Then temperature and respiratory rate should be commonly assessed by health workers given malaria and respiratory infections are the most common presentations and are relevant for the potential relation with hypoxemia why we chosed to include these variabels though not treating them as danger signs.
- Line 106 – “hospitals” is the incorrect term as participants were enrolled from hospitals and health centres. Better to refer to “health facilities”
  - This particular sentence refers to hospitals only as it intends to clarify that enrollment took place from the included hospitals’ outpatient departments only.

*Results*

- The results are presented in a series of 4 tables.
  Tables 1, 2, and 3 all contain data errors and only table 4 has any statistical analysis.
  Comments on individual tables are very limited and are restricted to the prevalence of the focus area of each table (hypoxemia, hypoglycaemia or danger sings) without any commentary on possible associations with or variations between subgroups ie age, gender, diagnosis or nutritional stuats.
  - Thank you for highlighting these issues. We have clarified tables and text as specified below including some additional comments on specific findings in tables.

*Table 1 – Baseline characteristics*

- A note should be added to indicated that children may have more than 1 diagnosis – there were 2,943 children in the study but 3,379 diagnoses.
  - This information has been added in Table 1
- There are 2 data errors - The number of mothers with secondary education who presented at a health centre should be 400 not 340 and the total number of children with a diagnosis of other infection should be 268 not 358 and the % needs to be adjusted from 12.2% to 9.1%
  - Thank you for identifying these errors in this table. The number of mothers with secondary/further education has been changed from 340 to 400. Regarding children with “other infection” as diagnosis, there was a typograhical error in the column for children presenting at a health center. It has been changed from 235 into 325. The total number and % is correct.
- A superficial analysis suggests that children who presented to the hospital were younger, had mothers with more education, a known nutritional status and more sepsis but fewer respiratory infections. Some statistical analysis is required to determine whether any of these are significant.
  - The difference in children seeking care at hospital vs at health centers were not part of our main objective why we chose not to assess or present this further. We believe one of the driving factors in where people seek care is the vicinity to the facility, something which we did not record in this study but may affect the findings (i.e. more educated/wealthy people tend to live more centrally where facilities are also located).

*Table 2 - Hypoglycemia*

- This table has 5 errors all in the final column for total numbers – I’m assuming this is the error as the numbers in the other columns do not match the totals.
- Total number of well nourished children should be 1669 not 1671
- Total number of children with missing nutritional status should be 925 not 949
- Total number of children with a temperature ≥37.5oC should be 890 not 892
- Total number of children with gastroenteritis is 187 not 188
  - Thank you for highlighting this. The reason for the errors was that the total number also inlcuded the children with missing glucose measurment. This has now been corrected and clarified in the table.
- Total number of children with a diagnosis of “other infections” is the correct sum of previous columns but is inconsistent with the number in Table 1 which was 268
  - Please see answer above regarding ”other infections” in table 1.
- Again there is no commentary on patterns with subgroups – children with moderate hypoglycaemia appear to be older, female, and to be sleepy and unable to drink
  - Please see added text on lines 317-319.

*Table 3 - Hypoxemia*

- This table has 7 errors all in the final column for total numbers – again I’m assuming this is the error as the numbers in the other columns do not match the totals.
- Total number of well nourished children should be 1472 not 1671;
- Total number of children with moderate malnutrition should be 130 not 154
- Total number of children with severe malnutrition should be 140 not 169
- Total number of children with missing nutritional status should be 893 not 949
- Total number of children with a temperature ≥37.5oC should be 818 not 892
- Total number of children with gastroenteritis is 155 not 188
- Total number of children with a diagnosis of “other infections” is 270 not 358 and this is also inconsistent with the number in Table 1 which was 268
  - Thank you so much for noticing this. Again, the above errors are due to that children with missing SpO2-value were included. Regarding the total number of children with ”other infections”; in Table 1 all children are included, but in table 3 only the children with a value of Spo2 are included. This is why the number of children with each diagnosis is not the same in every table.
- Again there is no commentary on patterns with subgroups – children with moderate hypoxemia appear to be younger males with convulsions or loss of consciousness
  - Please see added text on lines 348-349.

*Table 4 – Danger signs*

- There are no obvious errors in this table however a bit more information is needed – there were 848 children with danger sings which is 28.8% but how many had more than one danger sign, what were the number of children with danger signs in each age group.
  - As children with any danger sign should be referred for further care we have chosen not to report on numbers of danger signs given the limited space for the manuscript. Please see added info for age groups and danger signs in Table 4 and added text on lines 374-377 and 406-411.
- Whilst interesting I don’t believe that the temporal trends and Figures 1a and 1b add any value.
  - We have chosen to keep these as we believe it adds relevant information as discussed on line 556-560.
- Figure S1 needs a title and correction of the legend – IMCI refers to danger sings so relabel either as “IMCI danger signs” or just “”danger signs” rather than “IMCI”
  - Please note that the title of S1 Figure is at the end of the main manuscript document in line with PLOS author guidelines. To clarify, the legend has been changed to ”Danger sign” within the figure.

*Discussion*

- The discussion is reasonable however the sequence should ideally mirror the order in which data is presented in the results section i.e. hypoglycaemia then hypoxemia and then IMCI danger signs.
  - We have chosen to structure the discussion by first presenting our main findings and then discussing these findings in the subsequent paragraphs. We rather keep it in this way considering the overlaps in dicussion points for hypoglycemia and hypoxemia.
- The local findings are compared to a few studies of either in- or outpatient settings from LMIC however there is no comment on the age groups in these studies compared to the age group in this study. Presumably the outpatient studies using IMCI focus on the under-5 age group which is not the case in this study. So some comment is required on the age groups and how this may have affected the findings.
  - Thank you for highlighting the issue around including children above 5. We have added info on this in methods and results as mentioned above. We have also added text in the discussion, line 549-552 and 585-586. The prevalence of hypoxemia and hypoglycemia has not been well studied in general outpatients in LMICs, although we know of some data which should be published soon, we did not know of any already published comparable studies.
- There are a few “typos”
  - We have run a spell check on the manuscript.
- Lines 307, 309 and 323 refer to severe hypoglycaemia
  - The first two lines refer to the WHO definition and as WHO only defined hypoglycemia (i.e. no severe/moderate definiions) we chose to keep unchanged. ”Severe” has been added to line 476 where our results are discussed.
- Line 323 sign not sig
  - ”Sig” has been changed to sign
- Line 344 in a general population
  - This has been changed

*Limitations*

- This is adequately covered apart from the issue of using an assessment tool designed for children under 5 years of age on children aged 5 – 12 years.
- Please see this added to limitations lines 581-592.

*Conclusions*

The conclusion is appropriate

- Thank you

*References*

There is an extensive, comprehensive and up to date list of 44 references.
There are however inconsistencies in the structure of the references with some giving the first page number only and others the first and last pages.

- The references have been gone through one more time. References have been added using endnote and as in other publications in PLOS Global Health the pages are sometimes indicated with first and last page and for others with only first page. The EndNote style file “PLOS” is now used.
