# Supplementary material for: Hypoxemia, hypoglycemia and IMCI danger signs in pediatric outpatients in Malawi
Source: PLOS Glob Public Health. 2022 Apr 26;2(4):e0000284. doi: 10.1371/journal.pgph.0000284 (PMC10021275; doi:10.1371/journal.pgph.0000284)
Supplement: Attachment — Submitted filename: Response to Reviewers.docx [file pgph.0000284.s004.docx]

Dear Editors

Thank you for considering our manuscript “Hypoxemia, hypoglycaemia and IMCI danger signs in pediatric outpatients in Malawi” with reference numer PGPH-D-21-00696 for publication in PLOS Global Public Health. We have revised the manuscript according to the comments from the reviewers and editorial committee. Please see our responses to the issues raised listed in bullets below.

Best wishes,

André Thunberg, MD

**Journal Requirements:**

- Please review your reference list to ensure that it is complete and correct. If you have cited papers that have been retracted, please include the rationale for doing so in the manuscript text, or remove these references and replace them with relevant current references. Any changes to the reference list should be mentioned in the rebuttal letter that accompanies your revised manuscript. If you need to cite a retracted article, indicate the article’s retracted status in the References list and also include a citation and full reference for the retraction notice.
- Thank you for noticing this. The references have been reviewed again and the style file PLOS is used.
- Ref no 16 “Mini-review: Management of hypoglycaemia in children aged 0-59 months” has been updated to a new version that was published later.
- Ref no 17 “Relationship between hypoglycemia and mortality in critically ill children”. The title of the article was wrong and is now corrected.

**Additional Editor Comments (if provided):**

- There are a few spelling and grammatical errors in the manuscript. Please proofread prior to resubmission.
- A new spellcheck has been made
- Consider adding a comment in the manuscript regarding the missing nutritional status data and the differences between the healthcare center and hospital sites.
- A comment on the high proportion of children with missing nutritional status has been added in the discussion section, please see line 427-428. As the quality of nutritional assessment was not the focus of the study we have chosen not to add anything to the discussion regarding the difference in assessments between hospitals and health centers.

**Reviewer #2: GENERAL COMMENT**

The data errors have been corrected and the inconsistencies explained.
Comment has been added on the use of IMCI danger signs in the older, 5 – 12 year, age group and a comparison of findings between young and older age groups is now included.

- I believe that the principle of presenting words in full before using acronyms or abbreviations holds and applies to WHO which needs to be spelt out in full at the start of the document.
- WHO has been changed to World Health Organization in Abstract and upon first mentioning in the article.

The English is fair.

METHODOLOGY

- The authors have now provided a rationale for the use of IMCI danger signs in older children. However they still need to provide an explanation as to why they chose to supplement the 5 IMCI danger signs with temperature, respiratory rate and chest indrawing. They have provided this in their response to the reviewers but did not include this in the article. It needs to be included in the article.
  - Please see added info on row 171-174

The data has been cleaned and inconsistencies explained.

- Line 246 close the brackets (49.2%)
- This has been corrected.
- Line 321 “health worker” should be “research assistants”
- This has been corrected

DISCUSSION
This is reasonable and now includes comment on the different age groups.

LIMITATIONS
This has been expanded to include a comment on the use of an assessment tool designed for children under 5 years of age on children aged 5 – 12 years.
